# Electromyography Assessment During Gait in a Robotic Exoskeleton for Acute Stroke

**DOI:** 10.3389/fneur.2018.00630

**Published:** 2018-08-07

**Authors:** Ghaith J. Androwis, Rakesh Pilkar, Arvind Ramanujam, Karen J. Nolan

**Affiliations:** ^1^Human Performance and Engineering Research, Kessler Foundation, West Orange, NJ, United States; ^2^Children's Specialized Hospital, Mountainside, NJ, United States; ^3^Department of Physical Medicine and Rehabilitation, Rutgers–New Jersey Medical School, Newark, NJ, United States

**Keywords:** electromyography, rehabilitation, stroke, hemiplegic gait, robotic exoskeleton

## Abstract

**Background:** Robotic exoskeleton (RE) based gait training involves repetitive task-oriented movements and weight shifts to promote functional recovery. To effectively understand the neuromuscular alterations occurring due to hemiplegia as well as due to the utilization of RE in acute stroke, there is a need for electromyography (EMG) techniques that not only quantify the intensity of muscle activations but also quantify and compare activation timings in different gait training environments.

**Purpose:** To examine the applicability of a novel EMG analysis technique, Burst Duration Similarity Index (BDSI) during a single session of inpatient gait training in RE and during traditional overground gait training for individuals with acute stroke.

**Methods:** Surface EMG was collected bilaterally with and without the RE device for five participants with acute stroke during the normalized gait cycle to measure lower limb muscle activations. EMG outcomes included integrated EMG (iEMG) calculated from the root-mean-square profiles, and a novel measure, BDSI derived from activation timing comparisons.

**Results:** EMG data demonstrated volitional although varied levels of muscle activations on the affected and unaffected limbs, during gait with and without the RE. During the stance phase mean iEMG of the soleus (*p* = 0.019) and rectus femoris (RF) (*p* = 0.017) on the affected side significantly decreased with RE, as compared to without the RE. The differences in mean BDSI scores on the affected side with RE were significantly higher than without RE for the vastus lateralis (VL) (*p* = 0.010) and RF (*p* = 0.019).

**Conclusions:** A traditional amplitude analysis (iEMG) and a novel timing analysis (BDSI) techniques were presented to assess the neuromuscular adaptations resulting in lower extremities muscles during RE assisted hemiplegic gait post acute stroke. The RE gait training environment allowed participants with hemiplegia post acute stroke to preserve their volitional neuromuscular activations during gait iEMG and BDSI analyses showed that the neuromuscular changes occurring in the RE environment were characterized by correctly timed amplitude and temporal adaptations. As a result of these adaptations, VL and RF on the affected side closely matched the activation patterns of healthy gait. Preliminary EMG data suggests that the RE provides an effective gait training environment for in acute stroke rehabilitation.

## Introduction

Recovery of function post stroke is based on neural adaptation, and progressive task specific repetitive training based on the principles of neuroplasticity (1, 2). While major advances have been made in early intervention for the treatment of patients post stroke, the majority of survivors have residual mobility challenges and hemiplegia (3, 4). Hemiplegia typically manifests in pronounced asymmetrical deficits and is one of the most common disabling impairments resulting from stroke (5). Asymmetrical gait can be associated with muscle weakness, leading to inefficient ambulation, balance control challenges and risk of musculoskeletal injury to the non-paretic limb (6, 7). Task-oriented, high-repetition movements can improve muscular strength, motor control and movement coordination in patients post stroke (2). The task-specific training pertains to the training driven to achieve a functional task such as walking rather than focusing on minimizing an impairment (8, 9). In acute phase, traditional gait rehabilitation administered by a physical therapist is strenuous, inconsistent (in terms of movements generated) and less intense (in terms of number of steps). Integrating robotic exoskeleton (RE) technology into standard of care programs during the critical acute phase when the injured nervous system is highly plastic could maximize repetitive practice (9, 10), improve functional outcome measurements and provide quality gait training (10, 11). Programmable RE technology can also be used to advance progression during treatment and under the guidance of a physical therapist can emulate some features of manual assistance in a consistent and reproducible manner (2). The RE based training involves repetitive task-oriented (gait) movements and weight shifts to promote functional recovery. RE gait training may lead to changes in muscle activation as it provides task-specific movements to the lower limbs, increased step dosing and may provide a more symmetrical gait pattern (12).

An additional challenge in acute stroke is that many patients have a difficult time producing volitional movements that can be practiced repeatedly especially during the acute stage. In order to recover from physiological and functional lower extremity deficits, the task-related activities should include contributions from appropriate muscle groups during practice of these movements (13). Using an RE during gait rehabilitation in the acute phase may allow volitional muscle activation and improved phasic coordination (activation timing) during walking. However, the accuracy of these muscle contributions should be tracked. Surface electromyography (EMG) is one of the most effective, non-invasive tools which provides easy access to underlying neuromuscular processes that cause muscles to generate force, produce movement and achieve any functional task (14). During gait, EMG data reveals characteristic patterns of activation associated with each involved muscle in terms of onset timings, burst durations and levels of activations (15). These characteristic patterns significantly differ between healthy and pathological gait and this information can be used to assess the levels of improvement in muscle function, motor control, and neuromuscular adaptations post rehabilitation interventions. Bilateral EMG recordings of lower extremities can be further utilized to compare changes on the paretic side with respect to non-paretic side to assess inter-limb synchronization post RE intervention in individuals with stroke related hemiplegia.

To effectively understand the alterations occurring due to the RE, there is a need for EMG techniques that not only quantify the intensity of muscle activations but also quantify and compare activation timings for a single muscle during different gait training environments (e.g., overground or RE assisted). Although EMG amplitude is one of the most common variables reported in the literature (14, 16, 17), it does not distinctively provide temporal information (on–off timings). Particularly, in a cyclic activity such as gait, it is not only important for lower extremity muscles to produce activations but also activate them at the accurate time, especially for individuals with neurological disability such as acute stroke (16). In the post-stroke gait rehabilitation setting, the need to assess temporal information is even more apparent as muscle activation timing may be altered due to, (1) hemiplegia secondary to stroke and (2) the presence of a RE. The temporal features extracted from EMG data can allow the assessment of accuracy of participant's volitional contributions during training but also assess the modifications that the RE guided gait training may have. Several techniques have been used to extract the temporal information of muscle activations; however, their applicability in the domain of RE based gait training in acute stroke is limited.

The purpose of this investigation was to examine the applicability of a novel EMG analysis technique, Burst Duration Similarity Index (BDSI) during a single session of inpatient gait training in a RE and during traditional over ground gait training for individuals with acute stroke. EMG outcomes included standard measures of integrated EMG (iEMG) calculated from the root-mean-square (RMS) profiles, and a novel measure, BDSI (18) which quantifies the similarity between the two muscle activations by measuring co-excitation (common active regions) and co-inhibition (common inactive regions) during gait. Using iEMG and BDSI EMG analyses techniques, we hypothesized that the RE gait training environment will preserve the volitional neuromuscular activations in acute stroke. Volitional neuromuscular activations represent the residual post stroke muscle function during walking in the lower limbs. Our secondary hypothesis is that the RE gait training environment will change the activation timing of lower extremity muscles, measured by applying the BDSI technique, to match established normative healthy gait muscle activation timing patterns (15).

## Methodology

### Participants

Eligible participants were admitted to an acute inpatient rehabilitation facility, diagnosed with stroke (< 6 months), between the age 18 and 82 years and had to physically fit into the RE device (height between 1.5 and 1.8 m; weight < 99.7 kg). Five participants with acute stroke and unilateral hemiparesis (Age 51 ± 17 years; Height 1.7 ± 0.1 m; Weight 81.6 ± 3.6 kg; Time since injury 34.8 ± 34 days; Length of stay in acute inpatient rehabilitation 36 ± 24.6 days; Admission Motor Functional Independence Measure (FIM) 26 ± 4; three males, two females; two with right hemiplegia) were recruited for RE gait training during inpatient rehabilitation in conjunction with traditional therapy, Table 1. Participants had unilateral hemiplegia and lower extremity motor strength scores for all participants affected and unaffected side are presented in Table 2. All participants had: (1) no history of injury or pathology (unrelated to their stroke) within the last 90 days; (2) lower limb joint range of motion (ROM) within normal functional limits for ambulation; (3) no lower limb joint contracture or spasticity that limits ROM during ambulation; (4) sufficient strength of the contralateral limb to use an assistive device for ambulation; (5) upper body strength to balance with a walker or cane; (6) no skin issues that would prevent wearing the device; (7) stable blood pressure, with no diagnosis of persistent orthostatic hypotension, uncontrolled hypertension, or coronary artery disease. Participants were excluded if they had joint contractures of the hip, knee, or ankle that would prohibit the fitting of the RE, or concomitant medical conditions that would prevent inpatient gait training. Individuals were able to ambulate for 10 m with physical therapist assistance with and without the RE. All procedures performed in this investigation were approved by the Human Subjects Review Board and informed consent was obtained prior to participation in the study.

**Table 1:** Study participants' demographic information.

	**Type of assistive device**		**Gender**	**Age (years)**	**Height(m)**	**Weight (kg)**	**Affected side**	**Motor FIM**		**Length of stay (LOS) (days)**	**Time sinceinjury (days)**
**Participant**	**With RE**	**Without RE**						**Admission**	**Discharge**		
P1	RW	RW	M	73	1.70	80.6	Right	25	60	10	14
P2	QC	QC	F	30	1.63	87.8	Left	31	50	28	33
P3	QC, DW	NBQC	M	38	1.75	79.2	Left	20	57	49	94
P4	QC	QC	F	56	1.60	78.8	Left	27	42	13	19
P5	NBQC	NBQC	M	58	1.75	81.9	Right	27	66	11	14

*Type of Assistive Device: QC, quad cane; NBQC, narrow base quad cane; DW, dorsiflexion wrap; RW, rolling walker*.

*Motor Functional Independence Measure (FIM), Admission—The FIM Motor score measured at the time of admission to inpatient rehabilitation*.

*Motor Functional Independence Measure (FIM), Discharge—The FIM Motor score measured at the time of discharge from inpatient rehabilitation*.

*Length of Stay—Calculated as the time in days from admission to an acute inpatient rehabilitation facility to the date of gait testing*.

*Time Since Injury—Calculated from the date of the participants stroke (date of injury) to the date of gait testing*.

**Table 2:** Study participants' lower extremity strength scores.

**Joint movement**	**Affected side**					**Unaffected side**				
	**P1**	**P2**	**P3**	**P4**	**P5**	**P1**	**P2**	**P3**	**P4**	**P5**
Hip flexion	3+	1	3–	1	2–	4	5	5	5	5
Hip extension	3	1	3+	1	3	4	5	5	5	5
Hip abduction	3	0	2–	1	2+	4	5	5	5	5
Knee extension	5	2+	3+	2+	3	4	5	5	5	5
Dorsiflexion	3+	0	0	0	0	5	5	5	5	5
Plantarflexion	5	0	1	0	0	5	5	5	5	5

*P, participant; Strength measurements performed by treating physical therapist (maximum score was 5)*.

### Robotic exoskeleton (RE) device

**Figure 1:**
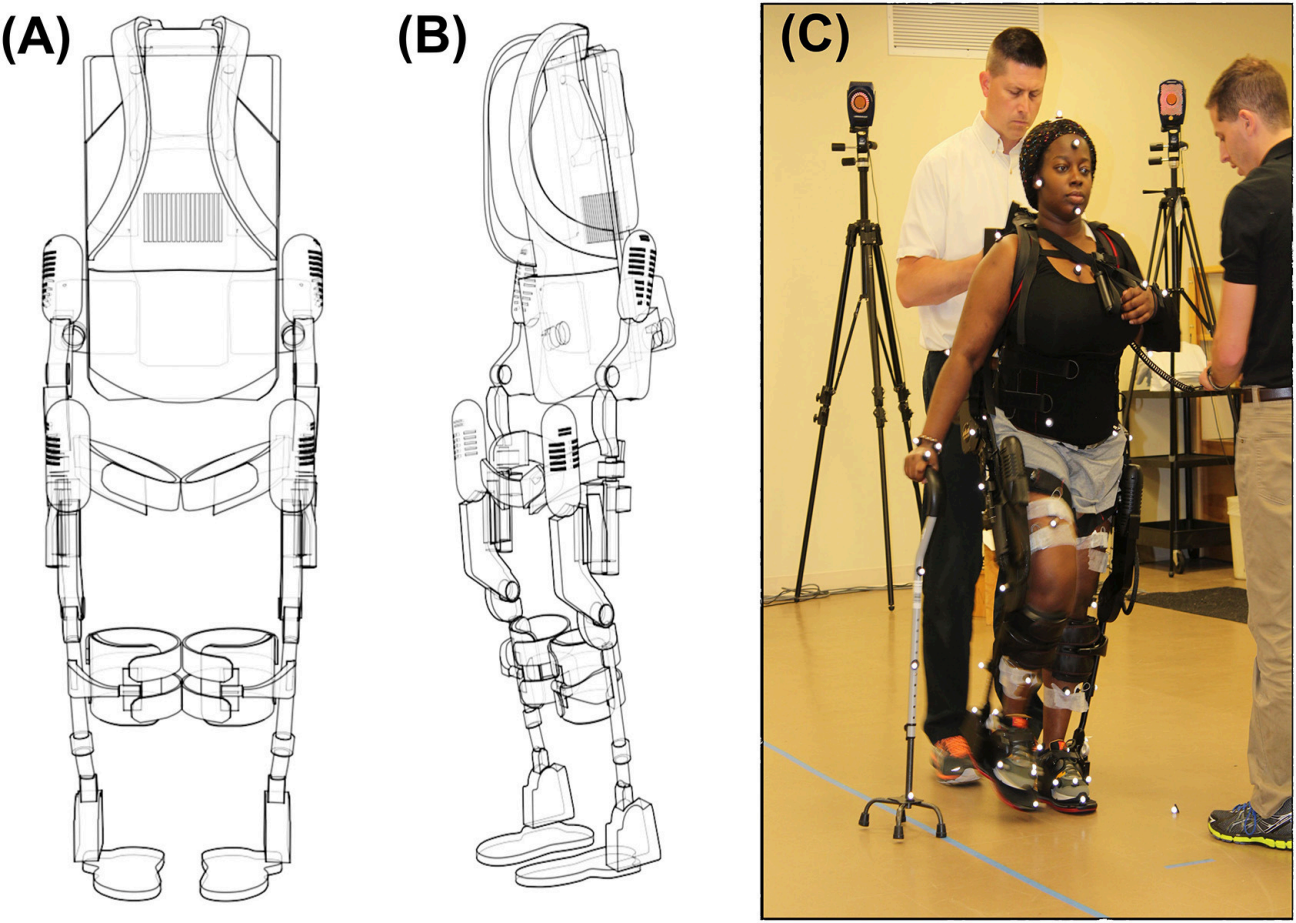
**(A)** Frontal view of the EksoGT, **(B)** Oblique view of EksoGT, **(C)** One representative participant in the commercially available RE device (EksoGT, Ekso Bionics, Inc. Richmond, CA, USA) during gait analysis and EMG data collection. (Media consent provided by the participant for publication).

Robotic gait training was provided to participants during stroke rehabilitation at an inpatient rehabilitation hospital through a commercially available FDA approved robotic exoskeleton (EksoGT, Ekso Bionics, Inc. Richmond, CA, USA). The RE is intended for overground gait rehabilitation under the guidance of a licensed physical therapist. The device provides motor assistance to patients by driving their angular joints of the lower extremity through a repetitive predefined trajectory to complete the gait pattern. The device is attached to the user with backpack style shoulder harnessing, a torso brace, affixed to the legs with upper thigh straps and shin guards on the shank, and a secure foot binding (Figure 1). The RE includes two powered joints (hip and knee) which provide bilateral angular motion and a passively sprung ankle joint with adjustable stiffness that provides resistance in the sagittal plane (dorsiflexion and plantarflexion). ROM provided at the ankle is from −10 to 20° dorsiflexion. The actuated ROM at the hip is −20 to 135° and the actuated range for the knee is 0 to 120°. Additional ROM is provided to assist with functions such as standing and sitting. The physical therapist can adjust the walking pattern (i.e., step speed and length) to facilitate progression and variable assistance to each leg. The control technique of the robot depends on participants shifting their center of mass (COM) laterally and forward onto the leading limb, while offloading the trailing limb during toe-off in preparation for the next step (RE step mode: ProStep+). The RE can be used in conjunction with assistive devices (cane, walker, hemiwalker, etc.).

### Experimental procedures

During a single session of inpatient gait training participants diagnosed with stroke performed walking trials on level ground at a self-selected pace under two conditions: (1) walking overground with an assistive device (similar to their traditional inpatient gait training environment) and (2) walking in the RE device with an assistive device. Table 1 identifies the type of assistive device used for each participant for ambulation with and without RE. Retroreflective markers were placed on anatomical landmarks and on the RE for all walking trials. EMG data were collected from six lower extremity muscles: tibialis anterior (TA); gastrocnemius (GA); soleus (SOL); rectus femoris (RF); vastus lateralis (VL); and biceps femoris (BF). During all walking trials, participants were allowed to stop and rest if necessary and research staff provided non-contact guarding for safety. The total duration of the experimental session was ~2 h. Kinematic data were collected at 60 Hz (Motion Analysis, Inc., Santa Rose, CA, USA), and time synched with wireless EMG data collected at 2,520 Hz (Noraxon, Inc., Scottsdale, AZ, USA). During initial post processing in Cortex software (Motion Analysis, Inc., Santa Rose, CA, USA) heel strike and toe-off gait cycle events were identified for all walking trials with and without RE. Heel strike and toe-off were determined based on the event of heel contact with the ground (or first foot contact in pathological gait) and the event of toe lift off of the ground (or the last foot contact with the floor in pathological gait) respectively. These temporal events were used for all subsequent EMG analyses to identify and normalize the affected and unaffected gait cycles as well as to identify and normalize the stance and swing phases of gait. An average of 11 gait cycles (minimum of 8 and a maximum of 14) were used for analysis of the with RE condition and an average of six gait cycles (minimum of 5 and a maximum of 6) were used for the without RE condition for each subject. EMG and temporal events were exported for further custom analysis in MATLAB (MATLAB R2014B, The MathWorks Inc., Natick, MA).

### Data processing and outcome measures

EMG data were band pass filtered (zero-lag, 4th order Butterworth; cut-off frequencies of 20 and 300 Hz) and notch filtered at 60 Hz. Filtered data were full wave rectified and root mean squared (RMS) with a 70 ms time window to smooth the data. For standardization, EMG data were normalized to 100% of a gait cycle based on the temporal events (heel strikes and toe-offs) extracted from the kinematic data. Each gait cycle (heel strike to heel strike) was subdivided into stance (heel strike to toe off) and swing (toe off to subsequent heel strike) phases. The stance and swing phases of the gait cycle were each normalized to 100% for standardization and to allow comparisons between and within subjects.

EMG outcomes included: (1) Amplitude analysis: integrated EMG (iEMG) and (2) Timing Analysis: Burst Duration Similarity Index (BDSI).

#### Integrated emg (iEMG)

iEMG is defined as the area under the curve of the rectified EMG signal. It is a parameter routinely utilized to compare EMG activation and is considered a measure of voluntary muscle drive. An increase in iEMG may be caused by an increase in firing frequency and the recruitment of additional motor units (19). In the current investigation, EMG data were segmented based on temporal events to indicate stance and swing phases. The iEMG during the normalized stance (0–100%) and normalized swing (0–100%) phases of gait were computed using a trapezoidal numerical integration in MATLAB as described in Equation 1 (20). Changes in iEMG were calculated for all collected muscles with and without RE. The calculated values of iEMG were averaged across participants and means and standard deviations were used for analysis.

(1) $$\begin{array}{l} \int_{a}^{b}{f\left(x\right)dx}^{\;}\approx \;\frac{b\;-\;a}{2N}\sum_{n=1}^{N}\;\left(f\left(x_{n}\right)\;+\;f\left(x_{n\;+\;1}\right)\right) \end{array}$$

*N* equals the length of the signal. In the integration process the term $\frac{b\;-\;a}{2N}$ is a representation of spacing between each point.

#### Burst duration similarity index (BDSI)

BDSI compares muscle activations (EMG “on”) as well as inhibitions (EMG “off”) and quantifies the match between the two EMG signals (18). For BDSI calculations, band pass filtered EMG data were normalized to 0–100% of gait cycles. For each participant, EMGs collected during all gait cycles for all walks were ensemble-average to get a single EMG representation for each muscle for each condition (with/without RE). Each EMG profile was processed using Teager Kaiser Energy Operator (TKEO). TKEO uses a sliding window approach to calculate the instantaneous energy changes with respect to neighboring samples. As a result, it amplifies the energy of the action potential spikes and differentiates between the relaxed and contracted muscle (21, 22). The baseline noise level for TKEO output was calculated and a threshold of eight standard deviations (SD) above the calculated baseline noise was determined. An EMG signal with amplitude above the calculated threshold for 10% of gait cycle was considered “ON” while amplitudes below the threshold for 10% was defined as the “OFF” period. Duration of 10% gait cycle was selected based on our previously reported data (18). Once the ON-OFF timings were determined for EMGs collected from each muscle during each walking condition, the BDSI between the two EMG signals, *s*_1_ and *s*_2_, of length *N* was determined in the following two steps,

- Create timing vectors as,
  On-timing: a binary vector of length *N* with 1 indicating simultaneous activation of *s*_1_ and *s*_2_ and 0 otherwise.
  Off-timing: a binary vector of length *N* with 1 indicating simultaneous inactivation of *s*_1_ and *s*_2_ and 0 otherwise.
- The BDSI, as a function of two EMG signals, *f*(*s*_1_, *s*_2_), is calculated as,
  (2) $$\begin{array}{l} BDSI=f\left(s_{1},\;s_{2}\;\right)=\;\frac{sum\left(On-timing\right)+sum\left(Off-timing\right)}{N}\times 100 \end{array}$$

BDSI were calculated by comparing EMGs collected from each muscle with and without RE as well as comparing to normative healthy adult gait activation. On-timing and off-timing vectors for healthy adult gait were generated from well-established normative data (15). Adult gait muscle activation timing information (in percent) presented in Perry et al. (15) was utilized as a healthy reference for the on-off durations during a normalized gait (100% gait cycle), and used in binary form (0–OFF, 1–ON) for comparison with the collected data (with and without RE) to compute BDSI scores.

**Figure 2:**
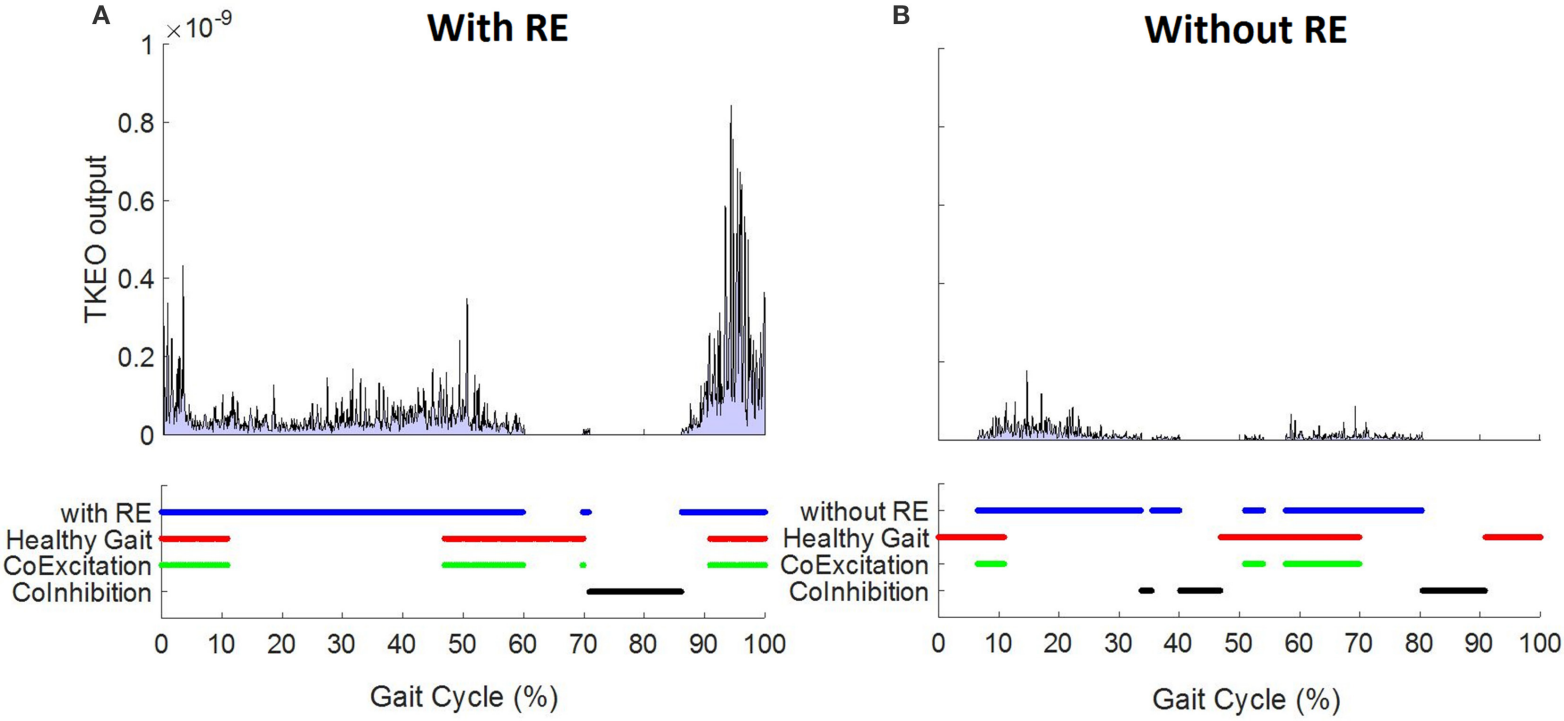
Left RF EMG onset detection using TKEO – 8 standard deviation threshold method for a 58 year old representative participant **(A)** with and **(B)** without RE.

Figure 2 demonstrates the EMG onset detection for a left RF muscle of one representative participant with and without the RE. Solid blue lines represent ON–OFF time points during a normalized gait (0–100% gait cycle) determined using TKEO processed 8 SD threshold method. Healthy gait is represented by solid red lines. Co-excitation (solid green) represents regions during a normalized gait when RF activation matched with the same for healthy gait. Co-inhibition represents the regions where both EMGs under comparison were inactive. Equation (2) was used to quantify the similarity based on co-excitation and co-inhibition in terms of BDSI.

### Statistical analysis

Paired sample *t*-tests were performed to determine if there were significant differences in mean iEMG and BDSI scores in two conditions: (1) walking overground with an assistive device (similar to their traditional inpatient gait training environment) and (2) walking in the RE device with an assistive device. Secondary analyses investigated if the RE gait training environment altered the activation timing of lower extremity muscles to match healthy muscle activation timing patterns. Paired sample *t*-tests were used to determine if there were significant differences between the affected and unaffected side while walking overground and in the RE.

## Results

### Electromyography amplitude analysis of lower extremity muscles

**Figure 3:**
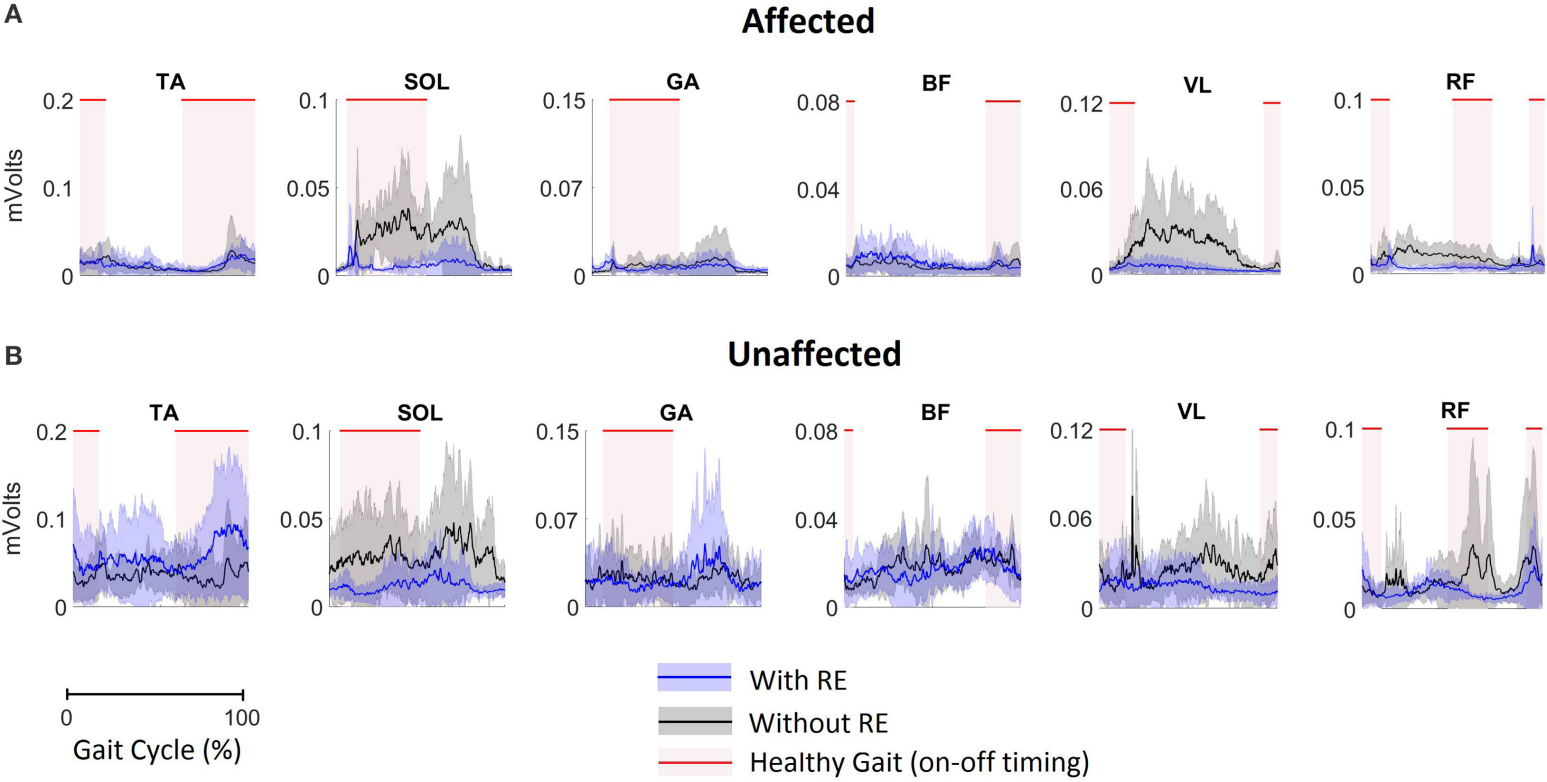
EMG activation of **(A)** the affected limb and **(B)** the unaffected limb for all participants (*n* = 5) during gait with and without RE. Shaded rectangular areas with red horizontal lines indicate when a muscle is active based on normative healthy adult gait, Perry et al. (15).

Mean EMG data demonstrate volitional although varied levels of muscle activations on the affected (Figure 3A) and unaffected limbs (Figure 3B), during gait with and without RE. These muscle activiations are characterized by variations in both amplitude and timing. In addition, the activations do not consistently correlate with the activation timing of healthy gait.

#### Bilateral mean iEMG changes with the RE

**Figure 4:**
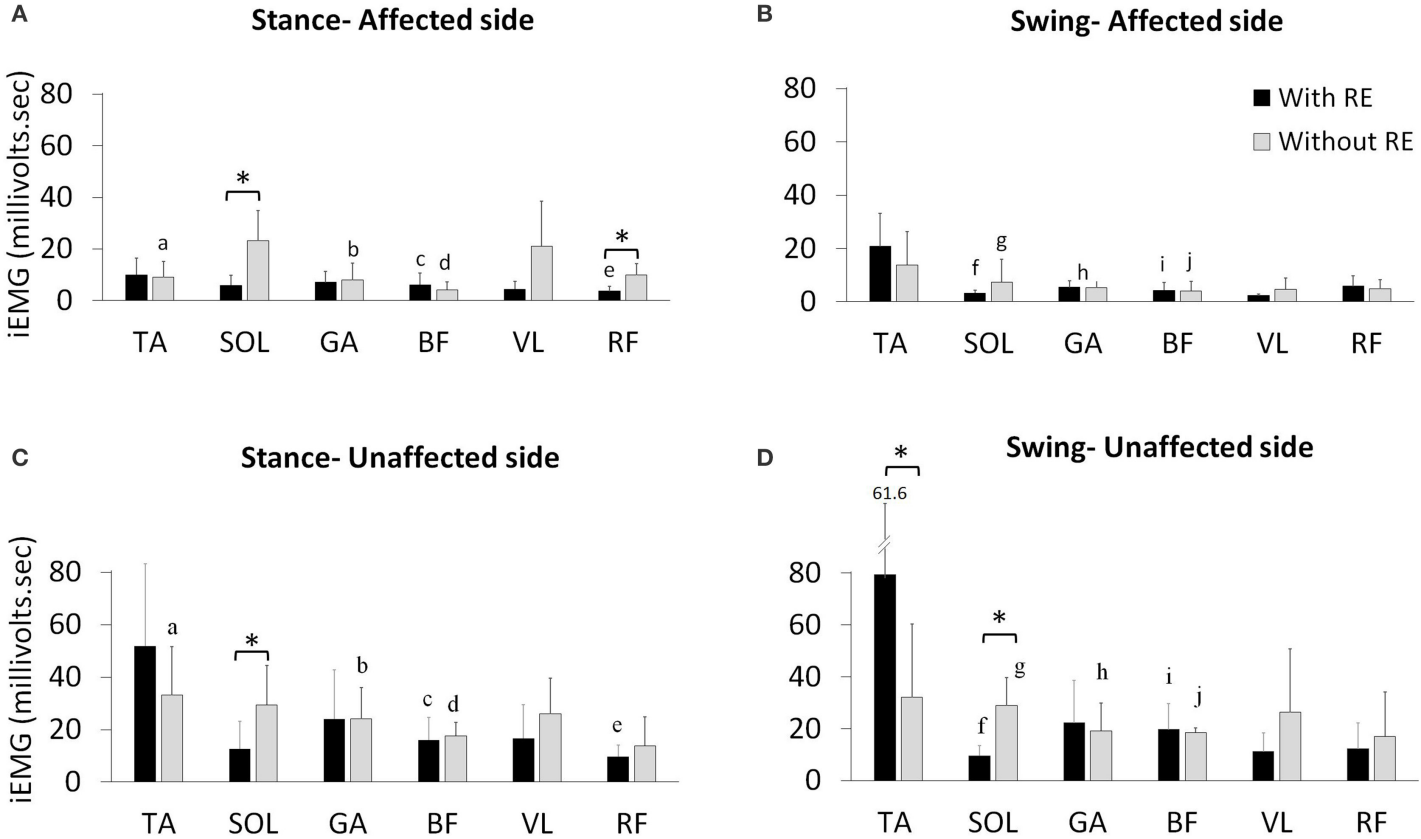
Mean iEMG of the TA, SOL, GA, BF, VL, and RF with and without the RE during the affected **(A)** stance and **(B)** swing phase of gait and the unaffected **(C)** stance and **(D)** swing phase of gait. *Within limb significant differences during stance and swing across gait training environments (*p* ≤ 0.05). ^a−j^The inter-limb comparisons during the stance and swing phases within the same gait training environment (with or without RE). a. TA affected vs. unaffected without RE during stance (*p* ≤ 0.05), b. GA affected vs. unaffected without RE during stance (*p* ≤ 0.05), c. BF affected vs. unaffected with RE during stance (*p* ≤ 0.05), d. BF affected vs. unaffected without RE during stance (*p* ≤ 0.05), e. RF affected vs. unaffected with RE during stance (*p* ≤ 0.05), f. SOL affected vs. unaffected with RE during swing (*p* ≤ 0.05), g. SOL affected vs. unaffected without RE during swing (*p* ≤ 0.05), h. GA affected vs. unaffected without RE during swing (*p* ≤ 0.05), i. BF affected vs. unaffected with RE during swing (*p* ≤ 0.05), j. BF affected vs. unaffected without RE during swing (*p* ≤ 0.05).

During the stance phase mean iEMG of SOL [with RE: 5.9 ± 3.9; without RE: 23.0 ± 11.8, *t*_(4)_ = −3.79, *p* = 0.019; effect size = 2.18, power = 94.61%] and RF [with RE 3.6 ± 1.8, without RE 9.8 ± 4.4, *t*_(4)_ = −3.92, *p* = 0.017; effect size = 2.34, power = 96.78%] on the affected side significantly decreased with RE, as compared to without the RE (Figure 4A), however, no significant differences were found for the other muscles on the affected side with the RE, as compared to without the RE (Figure 4A). During the swing phase, no significant differences were found for any muscles on the affected side with the RE, as compared to without RE (Figure 4B). There was an increase in iEMG of the TA during the swing phase on the affected side with the RE, but this change was not significant (*p* = 0.127).

During the stance phase mean iEMG of SOL [with RE: 12.5 ± 10.5; without RE: 29.4 ± 15.0, *t*_(4)_ = −3.91, *p* = 0.017] on the unaffected side significantly decreased with the RE, as compared to without the RE (Figure 4C). An increase in iEMG was observed for the TA during the stance phase of the unaffected side with the RE, as compared to without the RE, but this change was not significant (*p* = 0.145). During the stance phase, no other significant differences were found for the other muscles on the unaffected side with the RE, as compared to without RE. During the swing phase on the unaffected side mean iEMG of the TA [with RE: 79.4 ± 61.6; without RE: 32.0 ± 28.2, *t*_(4)_ = −3.00, *p* = 0.039] significantly increased while the SOL [with RE: 9.5 ± 3.8, without RE: 28.9 ± 10.8, *t*_(4)_ = −5.77, *p* = 0.004; effect size = 2.78, power = 99.39%] significantly decreased with the RE, as compared to without the RE (Figure 4D). During the swing phase no significant differences were found for the other muscles on the unaffected side with the RE, as compared to without RE.

#### Inter-limb iEMG comparisons without the RE

During the stance phase mean iEMG of TA [affected side 9.0 ± 6.2, unaffected side 33.1 ± 18.5, *t*_(4)_ = −3.84, *p* = 0.018], GA [affected side 7.9 ± 6.4, unaffected side 24.0 ± 11.8, *t*_(4)_ = −2.79, *p* = 0.049] and BF [affected side 4.1 ± 3.0, unaffected side 17.5 ± 5.2, *t*_(4)_ = −9.95, *p* < 0.001] significantly decreased without the RE on the affected side (Figures 4A,C). During the stance phase without the RE, no significant differences were found for the other muscles (Figures 4A,C). During the swing phase, mean iEMG of SOL [affected side 7.3 ± 8.4; unaffected side 28.9 ± 10.8, *t*_(4)_ = −3.74, *p* = 0.020], GA [affected side 5.1 ± 5.9, unaffected side 19.2 ± 10.6, *t*_(4)_ = −2.81, *p* = 0.048], and BF [affected side 4.1 ± 3.6, unaffected side 18.5 ± 1.7, *t*_(4)_ = −14.28, *p* = 0.002] significantly decreased without the RE on the affected side (Figures 4B,D). During the swing phase without RE, no significant differences were found for the other muscles on the affected as compared to the unaffected side (Figures 4B,D).

#### Inter-limb iEMG comparisons with the RE

During the stance phase, mean iEMG of BF [affected side 6.1 ± 4.5; unaffected side 16.0 ± 8.4, *t*_(4)_ = −4.62, *p* = 0.009] and RF [affected side 3.6 ± 1.8, unaffected side 9.6 ± 4.4, *t*_(4)_ = −3.03, *p* = 0.038] significantly decreased with RE on the affected side (Figures 4A,C). During the swing phase with RE, no significant differences were found for the other muscles on the affected as compared to the unaffected side (Figures 4A,C). During the swing phase, mean iEMG of SOL [affected side 3.2 ± 1.2; unaffected side 9.5 ± 3.8, *t*_(4)_ = −3.50, *p* = 0.024] and BF [affected side 4.2 ± 3.0, unaffected side 19.8 ± 9.8, *t*_(4)_ = −3.20, *p* = 0.032] significantly decreased with RE on the affected side (Figures 4B,D). During the swing phase with RE, no significant differences were found for the other muscles on the affected as compared to the unaffected side (Figures 4B,D).

In summary, the EMG amplitude analysis presented in terms of iEMG shows that the RE preserves the volitional muscle activation during walking. Moreover, while iEMG for TA is amplified, iEMG for SOL and RF is reduced during RE-assisted walking. To comprehensively understand these alterations, muscle activation timings were analyzed using BDSI.

### Burst duration similarity index (BDSI)

**Figure 5:**
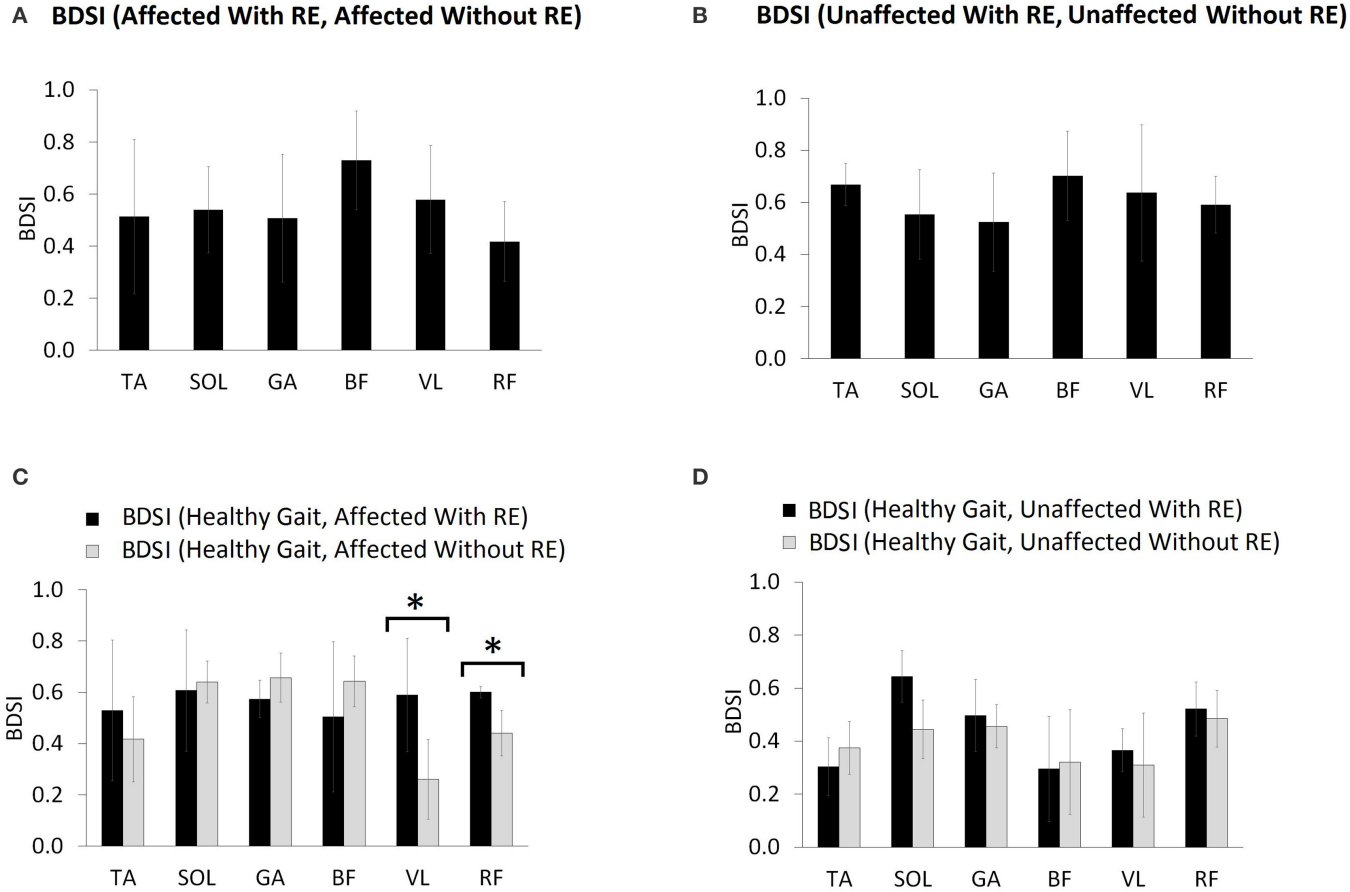
Mean BDSI calculated using Equation (2), by comparing **(A)** affected side with and without RE, **(B)** unaffected side with and without RE, **(C)** affected side compared to normative healthy gait muscle activation timing (15), with and without RE and **(D)** unaffected side compared to healthy gait with and without RE. The titles (for **A,B**) and the legends (for **C**,**D**) show the exact arguments used in the Equation (1) for computing BDSI. ^*^p ≤ 0.05.

BDSI scores were calculated for each muscle collected during normalized gait with and without RE and were averaged over all participants. Figure 5A shows the mean BDSI scores calculated by comparing muscle activation timings on the affected side with and without RE and Figure 5B shows the mean BDSI calculated by comparing muscle activation timings on the unaffected side. Higher BDSI scores (closer to 1) indicate the similarity in muscle activation timings with and without RE. In contrast, lower BDSI scores (closer to 0) in Figures 5A,B suggest the dissimilarity in muscle activation timings with and without the RE suggesting the alteration in the muscle activation during the RE assisted walking trials.

The RF muscle on the affected side showed the lowest BDSI score (0.42 ± 0.15) indicating the most dissimilarity in activation of RF muscle between with and without RE conditions (Figure 5A). The BF muscle was shown to produce the most similar muscle activations bilaterally (affected side: 0.73 ± 0.19, unaffected side: 0.7 ± 0.17) during both conditions (Figures 5A,B). Figure 5C shows the mean BDSI scores calculated by comparing muscle activation timings on the affected side to healthy gait muscle activations timings (15) during walking, with and without the RE. Higher BDSI values (closer to 1) suggest a closer match to healthy gait muscle activation timing (Figures 5C,D). The mean BDSI scores were higher for the TA (with RE: 0.53 ± 0.27; without RE: 0.42 ± 0.17), VL (with RE: 0.59 ± 0.22; without RE: 0.26 ± 0.16), and RF (with RE: 0.6 ± 0.02; without RE: 0.44 ± 0.09) on the affected side with the RE as compared to without the RE (Figure 5C). The mean BDSI scores on the affected side with the RE were significantly higher than without the RE for the VL [*t*_(4)_ = 4.6, *p* = 0.010; effect size = 2.06, power = 92.35%] and RF [*t*_(4)_ = 3.79, *p* = 0.019; effect size = 1.78, power = 83.91%]. These results suggest that utilization of the RE during walking significantly resulted in the temporal adaptations of VL and RF to more closely match healthy muscle activation patterns during gait. The posterior lower extremity muscles, SOL (with RE: 0.61 ± 0.24; without RE: 0.64 ± 0.08), GA (with RE: 0.57 ± 0.07; without RE: 0.66 ± 0.1) and BF (with RE: 0.50 ± 0.29; without RE: 0.64 ± 0.1) on the affected side showed a better match to the healthy muscle activation patterns without the RE when compared to with the RE, though no significant differences were found possibly due to the variability among the participants. On the unaffected side, the mean BDSI scores were higher for the SOL (with RE: 0.64 ± 0.1; without RE: 0.44 ± 0.14), GA (with RE: 0.5 ± 0.14; without RE: 0.46 ± 0.08), VL (with RE: 0.37 ± 0.08; without RE: 0.31 ± 0.2) and RF (with RE: 0.52 ± 0.1; without RE: 0.48 ± 0.11) with the RE when compared to walking without the RE, however, these differences were not significant for GA (*p* = 0.671), VL (*p* = 0.495), RF (*p* = 0.551), and near-significant for SOL (*p* = 0.054).

## Discussion

This investigation presented a traditional feature (iEMG) and a novel temporal feature (BDSI) of EMG data collected during RE-assisted walking in acute stroke. RE-assisted walking is a collective work of instrumentation power and varied levels of effort from a participant. Assessing these different levels of effort in terms of electrophysiological responses (via EMG) is essential for understanding the role of a user as well as the RE during gait training. In the acute phase post-stroke when the brain is highly plastic, these individual roles and the interaction between the user and the RE are essential in developing and evaluating the advanced training paradigms for gait rehabilitation. Further, the features extracted from the EMG data such as iEMG and BDSI allow the assessment of these individual roles and interactions during RE-assisted gait training. These features also help to understand if the neuromuscular adaptations occurring due to the RE are in accordance with the normative muscle activation patterns.

### Significance of iEMG and BDSI techniques

In this investigation, iEMG is presented as the amplitude measure and changes in iEMG may indicate variations in the levels of effort for a single muscle (15). iEMG, unlike root-mean-squared (RMS) or mean amplitudes, represents the neural drive to the muscle over a specified time. When iEMG is presented over an unspecified time interval it would simply represent the average over that unspecified time. In a cyclic activity such as gait, EMG profiles show characteristic patterns during different phases of gait. Therefore, utilization of iEMG for EMG quantification during gait phases may be more appropriate as these phases occur during a certain time duration in a gait cycle. Along with the amplitude parameter, we present a novel timing parameter, BDSI (18) to assess the temporal changes in muscle activation during gait, with and without the RE. Previous research has demonstrated the applicability of the BDSI algorithm to show a training effect on the TA muscle during hemiplegic gait post foot drop stimulator utilization (18).

TKEO (21, 22) based EMG processing was performed to improve the accuracy of onset detection and resultant BDSI scores on five additional lower extremity muscles (SOL, GA, BF, VL, and RF) including the TA. Although our onset detection component relies on a traditional approach of threshold detection (23), our BDSI calculation provides a novel way to compare EMG onset timings across many conditions. Since the BDSI calculation is performed on binary sequences (0: no activation, 1: activation), it not only allows for intra-limb (with and without RE), and inter-limb (affected vs. unaffected) comparisons but also provides the opportunity to compare to muscle activation timing patterns during healthy gait (15).

### Neuromechanical responses in RE environment

#### Amplitude adaptations using iEMG

We hypothesized that the RE gait training environment will preserve the volitional neuromuscular activations in acute stroke. iEMG analyses showed that muscle activation levels during RE gait training environment were maintained, reduced, as well as, enhanced, and there was no single common pattern associated with all tested muscles. This may suggest that the RE did not completely override the volitional muscle activations and the residual neuromuscular function post stroke was preserved and participants were actively engaged during the gait training. Although the inter-limb differences in EMG patterns post hemiplegia have been widely reported in the literature (24), they are not well established for acute stroke in a RE gait training environment.

**Figure 6:**
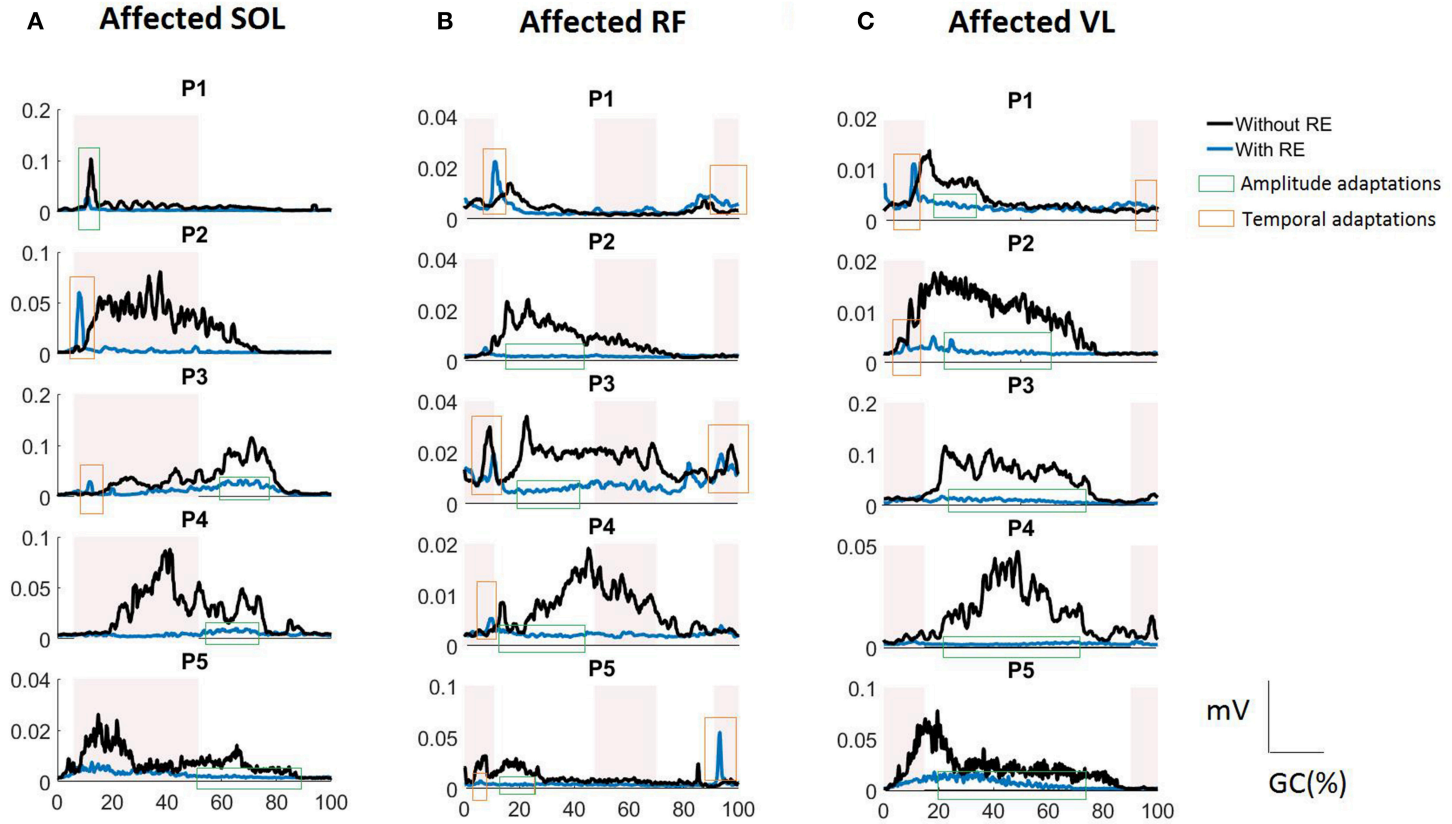
RMS EMG profiles for **(A)** SOL, **(B)** RF, and **(C)** VL of the affected side in the RE gait training environment for each study participant (P1 to P5). The shaded rectangular areas represent the periods of healthy muscle activations. Amplitude adaptations (shown as a green rectangle) represent the events where excessive muscle activation was reduced in the RE environment. Temporal adaptations (shown as a red rectangle) represent the events where the muscle activation was correctly time-shifted to the correct phase of the gait cycle in the RE environment.

In the current investigation we began to evaluate the effect of the RE on inter-limb activation. The iEMG analyses showed that for individuals post stroke in the RE environment the TA (during stance) and the GA (during stance and swing) muscles were no longer significantly different between the affected and unaffected side. This change could be due to the variability (shown by higher standard deviations) among the subjects (Figure 4) and may not be due to the reduced inter-limb differences in iEMG values for both muscles. There were increased TA activations bilaterally with the RE which may be due to several reasons: (1) the RE may promote the TA activation by providing a controlled trajectory and stable support during swing, allowing the muscle to activate in a stable environment; (2) TA may try to perform the ankle dorsiflexion against the foot strap during the swing phase, resulting in increased activation. There were significant reductions in iEMG values for SOL (stance), RF (stance) on the affected side with RE. This response seemed to be consistent for 4 out of 5 participants (shown as amplitude adaptations in Figure 6A for P2, P3, P4, and P5). During the stance phase with the RE, the significant decrease in the activation of SOL was observed potentially due to the quasi-static position of the sprung ankle joint at ~90°. It has also been reported that SOL muscle activation decreases when an external device is utilized such as an ankle foot orthotic (AFO) (25). In the presence of RE, the need to plantar flex against the ground for joint stabilization is reduced due to the weight bearing provided by RE, thus resulting in reduction of SOL activation, bilaterally. During the stance phase with the RE, the significant decrease in RF activation may be related to the reduction in “excessive” hip flexion at initial contact due to controlled trajectory guidance provided by RE. This was particularly apparent in affected RF profiles of participants P2, P3, and P4 (Figure 6B) where RF activations were reduced during 12–46% of a GC and 71–90% of a GC. Similar amplitude adaptations were observed for affected VL in the RE environment for all the participants, suggesting the consistency of these amplitude adaptation across the group.

Overall, iEMG analyses show the presence of active neuromuscular participation which may be essential for optimizing the rehabilitation outcomes during highly plastic acute phase post stroke. Ensuring that these adaptations are in accordance with the healthy muscle activation patterns is essential for effective gait training.

#### Temporal adaptations using BDSI

Our novel feature, BDSI allowed for this assessment with hypothesis that the RE gait training environment will alter the activation timings of lower extremity muscles to match the normative healthy gait muscle activation patterns. BDSI analysis showed that, in terms of activation timings, RF and VL on the affected side were significantly altered to match the normative muscle activation patterns in the RE training environment. EMG profiles showed that RF and VL, the muscles predominantly used for knee flexion/extension during walking had sustained contractions without the RE (Figures 6B,C) potentially due to the disrupted loading mechanisms on the affected side. While the amplitudes were higher for the RF and VL in the traditional gait training environment, BDSI analysis showed that these activations were characterized by inaccurate timings, compared to healthy gait (15). This was particularly apparent for P2, P3, and P4 as shown in Figures 6B,C (see the amplitude adaptations notations). In the RE environment the VL and RF had reduced activations (shown in Figures 6B,C) during the “off” periods (compared to healthy gait cycle) but preserved volitional activation during the “on” periods (shown as temporal adaptations in Figures 6B,C), closely matching the overall healthy activation timings during gait as evidenced by the significantly higher BDSI scores as a group (Figure 5C). These modifications could be due to the “powered” assistance provided during the loading response and knee extension during terminal swing or due to the possibility that the RE is programmed to follow a pre-determined trajectory that may not facilitate full knee extension and flexion. As surface EMGs were used in this investigation, RF may have recorded the crosstalk from vastii during loading responses (15). Hence, it may be difficult to identify the exact alterations occurring in RF muscle activation as a result of hemiplegia post stroke or due to the presence of RE. Apart from providing quantifiable comparisons for muscle activation timings with healthy muscle activation timings, BDSI feature also allowed for intra-limb comparisons with different gait training environments.

### Inter-subject variability

Although there were several improvements seen in terms of iEMG (bilateral VL, unaffected TA during stance) and BDSI (affected TA and SOL, GA, VL, RF on the unaffected side), these changes were not significant. This may be due to the variability in the data among the participants. In acute stroke, factors such as time since stroke and severity of the stroke can contribute to varied levels of residual function as well as responses to the RE intervention. All participants were currently admitted and participating in gait training at the same inpatient rehabilitation facility, and the motor FIM for all participants at admission was comparable (range from 20 to 31 points) indicating a similar level of motor impairment at admission. The 13 FIM motor items range from 13–91 points and rates an individual's ability to perform motor activities of daily living independently (26). All participants improved their FIM motor score from admission to discharge with an average improvement of 29 ± 11 points, the minimal clinically important difference (MCID) for the motor FIM is 17 points. While all participants had similar motor FIM scores at admission they were at different stages in their rehabilitation and recovery and time post stroke varied from 14 to 94 days and their length of stay at the time of gait testing varied from 10 to 49 days. Four participants were admitted to inpatient rehabilitation within 3–6 days post stroke, while one participant was not admitted until 45 days post stroke and this participant had the lowest motor FIM score at admission. All participants were able to successfully use the RE device with the assistance of a physical therapist as well as participate in inpatient gait rehabilitation. It is important to note that each participant progressed at an independent rate and may have been at a different timepoints in their recovery which will also have an impact on the EMG activation patterns during dynamic movements.

### Limitations and future considerations

This investigation presented an EMG technique and was not intended to comprehensively compare the efficacy of RE to traditional gait training and therefore only a single session was evaluated. Although we were able to demonstrate changes in activation of lower extremity muscles with the use of RE in acute stroke, our interpretations of the RE as a rehabilitation intervention is limited by the small sample size. Additionally, there may be variability due to the different levels of assistance provided by RE and physical therapists. Future work will include the kinematic indices which could be utilized to further understand the neuromuscular adaptations resulting due to RE in a larger sample.

## Conclusion

The EMG amplitude and timing analysis were presented to assess the neuromuscular adaptations resulting in lower extremities muscles during RE assisted hemiplegic gait post acute stroke. The RE assisted gait training environment allowed participants with hemiplegia post acute stroke to preserve their volitional neuromuscular activations during gait. The RE promoted activations of the VL and RF on the affected side to more closely match the activation patterns of healthy gait. The purpose of this investigation was to present an EMG technique and not to comprehensively evaluate the efficacy of RE over traditional standard of care. Instead, we demonstrated that patients were actively contracting and participated in both environments, therefore both environments represent potentially beneficial modalities in gait rehabilitation. Accurate understanding of the electrophysiological responses in individuals with stroke while walking is essential to develop and measure advanced training paradigms for gait rehabilitation. Preliminary EMG data suggests that the RE provides an effective gait training environment for acute stroke rehabilitation. Further, the combination of iEMG and BDSI techniques provides a comprehensive set of assessments to measure changes in muscle activation levels, excitation and inhibition during walking, with and without RE conditions in individuals with hemiplegia post acute stroke.

## Ethics statement

This study was carried out in accordance with the recommendations of Kessler Foundation Institutional Review Board with written informed consent from all subjects. All subjects gave written informed consent in accordance with the Declaration of Helsinki. The protocol was approved by the Kessler Foundation Institutional Review Board.

## Author contributions

KJN: contributed to the conception and design of the investigation; KJN, RP and AR: contributed towards data acquisition; AR: initial post-processing of the motion analysis data; RP and GA: performed the EMG analysis; All authors contributed to the interpretation of the data, manuscript writing and revision.

### Conflict of interest statement

The authors declare that the research was conducted in the absence of any commercial or financial relationships that could be construed as a potential conflict of interest.
